# National Rural Health Mission reforms in light of decentralised planning in Kerala, India: a realist analysis of data from three witness seminars

**DOI:** 10.1186/s12889-024-18181-x

**Published:** 2024-03-04

**Authors:** Hari Sankar D, Gloria Benny, Sreejini Jaya, Devaki Nambiar

**Affiliations:** 1 The George Institute for Global Health India, 308, Third Floor, Elegance Tower, Plot No. 8, Jasola District Centre, New Delhi, 110025 India; 2 Pallium India, Thiruvananthapuram, Kerala India; 3 George Institute for Global Health , University of New South Wales, Sydney, Australia; 4 Prasanna School of Public Health, Manipal Academy of Higher Education, Manipal, India

**Keywords:** Decentralisation, Community action for health, India, Health reform

## Abstract

**Background:**

The People’s Planning Campaign (PPC) in the southern Indian state of Kerala started in 1996, following which the state devolved functions, finances, and functionaries to Local Self-Governments (LSGs). The erstwhile National Rural Health Mission (NRHM), subsequently renamed the National Health Mission (NHM) was a large-scale, national architectural health reform launched in 2005. How decentralisation and NRHM interacted and played out at the ground level is understudied. Our study aimed to fill this gap, privileging the voices and perspectives of those directly involved with this history.

**Methods:**

We employed the Witness Seminar (WS), an oral history technique where witnesses to history together reminisce about historical events and their significance as a matter of public record. Three virtual WS comprised of 23 participants (involved with the PPC, N(R)HM, civil society, and the health department) were held from June to Sept 2021. Inductive thematic analysis of transcripts was carried out by four researchers using ATLAS. ti 9. WS transcripts were analyzed using a realist approach, meaning we identified Contexts, Mechanisms, and Outcomes (CMO) characterising NRHM health reform in the state as they related to decentralised planning.

**Results:**

Two CMO configurations were identified, In the first one, witnesses reflected that decentralisation reforms empowered LSGs, democratised health planning, brought values alignment among health system actors, and equipped communities with the tools to identify local problems and solutions. Innovation in the health sector by LSGs was nurtured and incentivised with selected programs being scaled up through N(R)HM. The synergy of the decentralised planning process and N(R)HM improved health infrastructure, human resources and quality of care delivered by the state health system. The second configuration suggested that community action for health was reanimated in the context of the emergence of climate change-induced disasters and communicable diseases. In the long run, N(R)HM’s frontline health workers, ASHAs, emerged as leaders in LSGs.

**Conclusion:**

The synergy between decentralised health planning and N(R)HM has significantly shaped and impacted the health sector, leading to innovative and inclusive programs that respond to local health needs and improved health system infrastructure. However, centralised health planning still belies the ethos and imperative of decentralisation – these contradictions may vex progress going forward and warrant further study.

## Introduction

‘Community participation was established as one of five key principles to achieve Health for All by 2000 in the Alma Ata Declaration of Primary Health Care [1]. In recent times, momentum around social participation for health is growing [2], and with it, demand for more significant documentation of and evidence about the contexts in which people have mobilized for the cause of health, how, and to what effect.

In India, various collectives, individuals, and institutions set the stage for community action by institutionalising governance mechanisms through decentralisation reforms of the 73rd and 74th constitutional amendments in the early 1990s. The amendments ensured the creation of elected governments at three levels, i.e., the grama (village level), block, and district, ensuring one-third reservation of all seats and chairperson positions to women and those from “marginalised sections” of society [3]. Decentralisation thus changed the then-existing top-down approach of governance by transferring power to an elected Local Self Government (LSG) or panchayat, giving them greater political authority and administrative responsibilities. Decentralisation and participatory planning are among various other forms of social participation in health, like community-based monitoring, civil society-led service delivery, frontline health worker programs and more that have varying histories and trajectories across Indian states [4, 5].

Preceding the 73rd and 74th amendments in the southern Indian state of Kerala was mass mobilization carried out by various civil society organizations, which fostered literacy and scientific temper and later sought to nurture economic development and micro-enterprise level, evolving into the much-lauded People’s Planning Campaign (PPC) by 1996 [6, 7]. Under the PPC, the state devolved functions, finances, and functionaries to LSGs to activate participatory planning [8, 9]. In the health sector, the PPC created an entry point for the incubation of innovative health programs at local levels, mobilizing voluntary resources to support them, and overall, providing spaces and support for people to voice their perceived health needs through a participatory mechanism [10]. In some cases, this also raised people’s expectations of the system, creating conflict and some dilemmas for health system responses on the supply side [11].

Alongside these developments, at the national level, the Ministry of Health and Family Welfare 2005 introduced a series of architectural reforms into the rural health system; collectively organised under the banner of the National Rural Health Mission (NRHM) which was later renamed the National Health Mission (NHM). As our study traces the program from its beginning to recent times, we use the acronym N(R)HM to denote the program. N(R)HM included various forms of community participation, or “communitization,” of health and health services [12, 13]. Communitization was seen as a strategy to improve public health system performance, reduce inequities, and increase a sense of community ownership and system accountability [14]. The N(R)HM program envisaged community action through an active female frontline Community Health Worker (or ASHA) program, grassroot level community participation, and monitoring mechanisms like Rogi Kalyan Samiti (Patient Welfare Committee), Village Health and Sanitation Committees (VHSNCs), and through the involvement of NGOs in health service delivery [15].

A fair bit of documentation – much needed - of the PPC and decentralisation in Kerala has been undertaken [8, 16–18]. However, less emphasis was placed on this canon in the (more recent) period after N(R)HM entered the fray in Kerala. The particular history of interaction between decentralisation reforms stewarded by state government based on bottom-up planning and N(R)HM reforms- a union government program with top-down planning in Kerala may offer unique insights for the rest of India and other Low and Middle Income Country contexts. Moreover, documenting history like this makes demands of method beyond the typical approaches like case studies or interviews. Given that history itself is co-constructed, we sought to privilege the voices and perspectives of those directly involved with the history of decentralisation and N(R)HM through the method of Witness Seminar (WS) and made this available for public record and learning [19]. Following this exercise, in this paper, we sought to draw out key lessons from this history for community participation and health systems strengthening. More narrowly, our question was: in what contexts and through what mechanism did Kerala’s decentralisation reforms and N(R)HM reforms occur, and what impacts did they have on the health system?

## Methods

We carried out three virtual WS between June and September of 2021. The WS is an oral history technique where the emphasis is placed on a period set of developments in history, and those who bore witness to and were part of this history together detail their experiences and significance as a matter of public record [20]. More details about the methodology are available elsewhere [19]. The WS series and handbook created by a prominent Indian research and advocacy group, SAATHI has guided and informed the conduct of this WS series in Kerala [21, 22].

Persons involved with the PPC, N(R)HM, academia, elected representatives, and officials from the health department were invited to participate in the WS. Seminars were recorded, transcribed in English and Malayalam, and sent to participants for approval. Detailed annotations were carried out on transcripts, and additional translations were made as appropriate. WS transcripts were analysed using Atlas.Ti 9 software [23]. Initially, inductive thematic analysis of transcripts was carried out by three researchers in discussion with a fourth. Codes were indexed and grouped, and analytical approaches were explored, based on which a realist framework was chosen. Realist approaches have been used to look at complex health systems phenomena and qualitative research [24, 25], as well as community action for health in particular [26–28], and were considered uniquely appropriate for our analysis. We also aligned the realist philosophy of “subtle realism” that privileges the representation of reality, rather than the pursuit of a single truth (or in this case, history) [29]. Guided by Mays and Pope [29] three researchers, a male research fellow, a female research assistant, and a female research consultant, charted our theme groups under Context, Mechanisms, and Outcomes (CMOs) to develop configurations drawing from the data. The analysis was reviewed by a senior female health systems researcher. This took multiple rounds of discussion, revision, and re-charting, based on triangulation across witness seminars and participants, attention to reflexivity (and our understandings of these histories and relationships with the witnesses) which in turn sharpened our attention to negative cases. We made sure we were fair dealing, and were not biased by individual alignment with witness accounts and our role in each seminar (e.g. one of us was moderator). We eventually arrived at two broader sets of CMO configurations that we lay out in our results.

We obtained informed consent from all participants while conducting WS. The study was approved by the Institutional Ethics Committee of the George Institute for Global Health (27/2020).

## Results

Three witness seminars were carried out with a total of 21 participants, of which five were female (see Table 1).

**Table 1:** Participant characteristics

Participant type	Male	Female
Senior health administrators (Policymakers)	3	0
State program functionary N(R)HM	3	1
Elected representative	1	1
Medical officer	1	2
Academic	4	1
Frontline public health staff (FPHS) member	4	0
TOTAL	16	5

*Note* The profiles included persons who were retired from service and those who remained in service

**Fig. 1:**
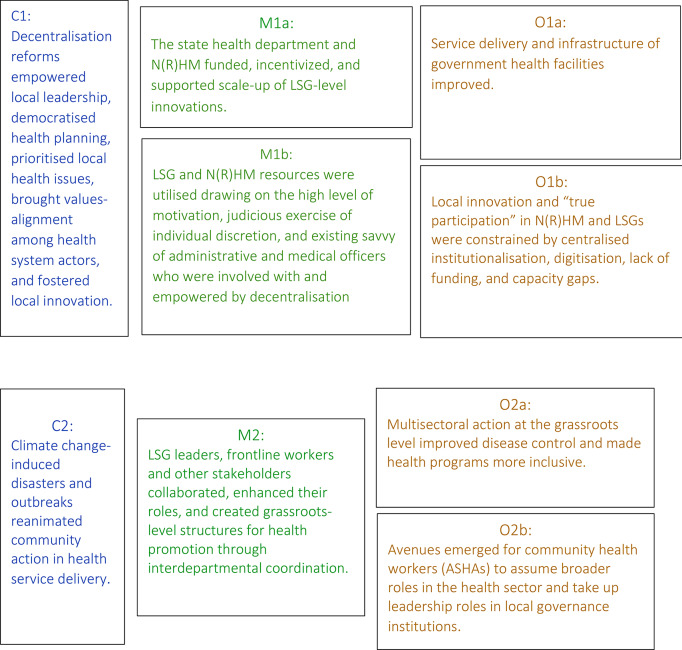
Contexts, mechanisms, and outcomes associated with state level decentralisation reforms in Kerala and national health reforms in India

Our analysis revealed two sets of CMO configurations that describe the interaction between decentralisation efforts and N(R)HM in Kerala (see Fig. 1).

### C1: Decentralisation reforms empowered local leadership, democratised health planning, prioritised local health issues, brought values-alignment among health system actors, and fostered local innovation

Witnesses observed that the provision of functions, functionaries and funds from the state plan fund to LSGs as part of decentralisation reforms in Kerala 1996 onwards empowered them with decision-making power, financial resources, and human resources to facilitate local planning and program implementation. The provision of untied funds, i.e.,funds transferred to local governments with freedom for local planning, was crucial in reposing authority and power in local leaders.The Kerala Panchayati Raj Act 1994 and Kerala Municipality Act of 1994 also ensured that partial control of local level public institutions like schools, primary health centres, agriculture offices, and the like were transferred to LSGs. Maintanence and (partial) administrative control of these institutions were entrusted to LSGs while recruitment and ownership of buildings remained with parent departments. The PPC process helped to enhance the capacity of local governments to plan and implement development projects within their jurisdictions. This environment fostered the rise of several innovative programs to address locally prioritised health problems. A senior policymaker detailed the importance of fund provision and autonomy in decentralised planning as follows:

The most important feature of Kerala’s decentralisation is the practically untied grant devolved to the local governments in the form of a development fund which constitutes more than 25% of the state’s plan outlay. Since the development fund and maintenance fund are relatively untied, there is competition for resources among institutions and sectors. This motivates many institution heads to propose new ideas and improve performance to attract more funds. This authority through the fiscal route has been more effective in practice than the formal powers. (WS-1 Policymaker-1 male)

Along with empowering the LSGs with resources, the PPC, supported by the state government mobilised communities, provided technical handholding to design solutions and channelised community action for local health planning. The campaign helped in improving the awareness of the community on health issues and brought them closer to the LSGs and health department officials. A Frontline Public Health Staff member (FPHS) from the Department of Health recalled,

It was that people were able to identify their problems on their own, and they were able to find out a technically feasible and economically viable solution with the help of their elected representative and with the experience of a local expert such as a health worker. These great models were made when problems were identified, prioritised and LSGs addressed these problems based on that priority. The unique palliative care projects etc. are considered as such models. These … have been the byproducts of the freedom offered by decentralised planning. (WS-2 FPHS-1 male)

The empowerment of LSGs facilitated innovations at different scales. A senior health department administrator stated how decentralised planning initiated and continues to support large-scale programs, saying that “decentralisation has given avenues for innovations in health care. And in fact, community-based pain and palliative care program and later the NCD program started at the panchayat level, especially in northern Kerala – we consider it an innovation.” (WS-1 Policy maker-2 male).

There were several such innovations; a senior academic associated with the decentralisation process from its early days recollected some of the innovative projects planned and operationalised by LSGs for disease control locally, targeted to address specific health needs.

I remember the fish farming project in Kasaragod. I remember distributing guppy fish in an initiative against mosquito-borne diseases in Pilicode. Similarly, installing and finding budgets for mosquito screens in the hospitalwindows as a part of the Kasargod district panchayat’s initiative. I remember, Kudumbashree [self help group] workers in Kozhikode, Thrissur and Thiruvananthapuram did not have personal protective equipment while they were engaged in waste management. I remember giving them awareness and making it into a project[to provide PPE kits]. (WS-3 Academic-1 male)

### M1a: The state health department and N(R)HM funded, incentivised, and supported scale-up of LSG level innovations

The successful implementation of LSG-initiated projects in health and health-related areas post decentralisation in different panchayats of the state were noticed by the state health department and N(R)HM. State officials recognised the potential of these projects and scaled up selected LSG projects to the entire state. A frontline public health official in the department said:*”* These [palliative care and NCD control projects] were initially started by the LSGs. Later, the government realised the potential and importance and took it up as their major state (health) program.“(WS-2 FPHS-2 male) Another senior administrator from the health department commented on the role of N(R)HM in providing resources for scaling up these projects, saying that “when [the] Palliative care program was taken up for state-level implementation in southern Kerala, it was difficult to expand the program and hence it was taken up as an NHM component.” (WS-1 Policymaker-2 male).

The PPC ensured that LSGs would be the focal point of development at the panchayat level but the resource pool for the developmental agenda led by LSGs was distributed across multiple stakeholder departments like veterinary science, education, agriculture, and health. To facilitate and incentivise increased fund allocation to the health sector, NHM started the Comprehensive Health Program (CHP), Arogya Keralam Puraskaram (later renamed the Ardra Keralam Puraskaram), and Arogyadarshan. CHP was designed to train health officials in preparing project submission plans and familiarise them with rules and guidelines regarding fund utilisation. An FPHS who was part of the CHP program recollected:

First, we were worried about audit issues. Implementing officers were given training and were empowered. It was the time when the Sulekha software [developed by the LSG department for monitoring LSG program plans] was launched. All the implementing officers and doctors in the health department were given training on Sulekha. And some basic lessons on plan formulation, financial guidelines etc… We could say that we were utilizing NRHM funds because there is a social development fund under NRHM. (WS-3 FPHS 1 male)

The Ardra Keralam Puraskaram is a cash prize given by the state’s Chief Minister to the LSGs that make significant investments from the annual plan fund towards the health sector. This award also recognizes the LSGs implementing the most innovative health or health-related projects each financial year. Arogyadarshan was a TV program on national television that featured the LSGs whose best practices in the health sector were awarded the Ardra Keralam Puraskaram.

A senior N(R)HM official attributed the increased fund allocation to the health sector by LSGs to these programs. He said: “The number of projects taken up for the health sector steadily increased every year. From around 2%, [of LSG plan fund] around 8–9% was set aside [now] for this sector. The good thing is that we were able to get more projects and maintain the quality of those projects.” (WS-3 N(R)HM official-1 male).

### M1b: LSG and N(R)HM resources were utilised drawing on the high level of motivation, judicious exercise of individual discretion, and existing savvy of administrative and medical officers who were involved with and empowered by decentralisation

In most cases, a medical doctor heading a public facility would serve as the implementing officer for LSG-funded health programs. This person was seen to be a key factor by our participants for “effective” and “creative” utilisation of various funds available through N(R)HM and LSGs. Multiple witnesses mentioned the importance of the motivation and “self-efficacy” of these officers in the effective implementation of health initiatives at panchayat. A scholar-professor who taught medical students for many years in a teaching hospital mentioned that:

Initially (post PPC), there was some conflict between the doctors and the local self-government bodies and the politicians, but soon such doctors realised the benefits of cooperation with local self-government bodies. Particularly, those doctors with a background of political or social activism in their student days or after could appreciate the merits of this system and make many meaningful transformations.(WS-1 Academic-2 male).

A former Panchayat President noted that the motivation and commitment of implementing officers influenced their ability to understand and interpret the official guidelines of implementation. Kerala’s planning process has always been stringently monitored, with regular financial audits. The implementing officer is responsible for verifying and maintaining the necessary documentation. While more broadly, this would tend to incentivise projects that would be free of audit objections, some officers found ways to work creatively with LSG members and incorporate innovation, as explained by another witness:

In my experience, most often the [planning] guidelines were not in our favour. There was a phase when the implementing officers limited their activities within those guidelines; There is a tendency to do projects [which do not create] audit objections, even today. But when we got officers who could read between the lines and medical officers and health workers who worked side by side, we were able to plan projects. (WS-2 Elected representative-1 female)

### O1a: The service delivery and infrastructure of government health facilities improved

Local self governments took over the ownership of public health institutions in the state, invested in maintaining and improving the infrastructure and service delivery of their respective institutions. The quality of service delivery in government health facilities improved significantly after the launch of PPC in the mid-nineties. In addition to maternal and child health care, to which the public system traditionally catered, other locally relevant health issues started to receive attention. A policymaker remarked:

Both local governments and the health department realized the importance of cooperation and consciously pushed for it. Local governments, especially the grama panchayats, took pride in improving the facilities of the hospitals under their control, outreach improved, and Non-Communicable Diseases (NCD) and new Communicable Diseases became the focus of action. (WS-1 Policy maker-1 male)

This was corroborated by an FPHS working in the department of health who said that “another important thing is, local government at different places managed additional resource mobilisation to improve basic infrastructure at the primary health centre, community health centre, and Taluk Hospital.” (WS-1 FPHS-3 male).

Since 2005, the National Rural Health Mission (N(R)HM) has played a crucial role in supplying essential Human Resources for Health (HRH) to execute healthcare initiatives throughout the country. The Department of Health Services Kerala, leveraged this opportunity; by 2022, nearly a quarter of the staff working in clinical and nonclinical cadres in the government health system were employed through N(R)HM. A senior N(R)HM official who works in the state program management support unit noted: “ Nearly 12,000 people today are working under N(R)HM. The [Health] Department has around 45,000 staff.” (WS-3 N(R)HM official-2 male).

While LSG support improved the basic infrastructure of government health facilities, N(R)HM, along with the financial support to improve infrastructure, introduced quality standards and accreditation to improve service delivery. Quality standards for care were developed and government health institutions were encouraged to improve the quality of service delivery to meet the prescribed standards for care. The National Accreditation Board for Hospitals & Healthcare providers (NABH), started by the Quality Council of India, was instrumental in benchmarking the quality of care delivered through public and private health facilities in India. The digitisation of health data also started during the N(R)HM phase. A medical officer from Alappuzha noted: “The change of face of all the PHCs was facilitated by N(R)HM… The idea of electronic health records was implemented through N(R)HM.…. We were able to do a lot of quality (improvement) programs at < name of facility>.” (WS-3, Medical officer-1 female) A senior health administrator also elaborated on the health care quality improvement initiative by N(R)HM in Kerala, linking it to the National Quality Assurance Standards (NQAS) for health care prescribed by the national government.

It was during the N(R)HM time, that we started the quality improvement programs in an organized manner. We posted the quality officers and biomedical engineers to achieve NABH accreditation for the institution. The programme is continuing, now we have more than 120 NQAS accredited healthcare institutions. (WS-1 Policy maker-2)

### O1b: Local innovation and “true participation” in N(R)HM and LSGs were constrained by centralised institutionalisation, lack of funding, and capacity gaps

Both LSGs and N(R)HM had the flexibility to design new programs tailored to local health needs initially. Gradually, with the scale-up of the programs, the human resource and institutional costs of established running programs started taking the lion’s share of the budget. This restricted the scope for further innovation as the total budget allocation from N(R)HM and state government stagnated for many years until in 2021, the Fifteenth Finance Commission made allocations directly to LSGs to improve health infrastructure and recommended that state governments transfer central funds directly to LSGs as well.

Reflecting on the period prior to 2021, a senior official from N(R)HM commented that, over time, the scope for bottom up planning through the N(R)HM budget has become limited.

Every year, we (N(R)HM) have to prepare a project implementation plan (PIP), which has to be given to the Government of India. So mandatory activities will come up in the first place. There are many such things that we cannot forgo when it comes to PIP. As a result, the remaining budget will be limited. And with that, when we have some untied funds, the overall kitty will get covered. So, we have no other choice but to say that it is a top to bottom approach now, unfortunately. (WS-3, N(R)HM official-2 male)

Over the years, our participants observed a decline in people’s participation in the planning process. The planning through *gram sabha* (village planning committees) was reportedly not as rigorous as it used to be in the time of PPC in 1996. After the introduction of digitalisation of project submission, community involvement became limited and the process, mechanical. It was reported that officials were limiting the annual proposals for LSG projects to only include mandatory initiatives. These projects were initially introduced as LSG projects and have received recognition, leading to their implementation in all LSGs in Kerala as mandatory projects, ensuring their sustainability. An academic with long-standing ties with LSGs and association with LSG project planning voiced this concern:

Since 2010, people’s participation in planning has been declining; by the time the 11th plan got over in 2010 with the introduction of computers, things had become like complete packages. Earlier when we write up a project, there’s a clear background to it, who are the beneficiaries, what is exactly happening, how is it monitored etc. That’s my personal opinion, and many others have also shared it. Since things got computerised in the 12th plan, the participatory character was lost. (WS-3 Academic-1 male)

A former LSG member expressed concern that recent developments in decentralised planning were leading to centralised planning, reducing the operational autonomy of LSGs. The question of “true power” remains a concern for LSGs as the health department still owns the lands where health institutions are located and handles the recruitment and payroll of health department staff. The guidelines for LSG program audits are also developed at the state level, which in a sense hinders the autonomy of LSGs:

When I was the chairperson of the Panchayat Welfare Committee last time, we facilitated a “BUDS” centre for people with different abilities [sic.]. Similarly, a multipurpose yoga and fitness centre for women. It was part of a broad project for intervention in the women’s health sector. Since all this was built in the PHC compound, there was an audit objection against the doctor worth some Rs. 60 lakhs alleging that there was no permission sought from the department. What does this mean? Even with the decentralisation of power and we say that power is with the panchayat etc., who owns the land? It is owned by the department. (WS-2, Elected representative-2 male)

A medical officer from the Department of Health pointed out a challenge regarding the level of acceptance of decentralisation and PPC among implementing medical officers in the state, which in turn hindered the implementation and sustainability of health projects using LSG funds in the state:

Unlike what we tend to think, it is not a rosy picture in Kerala. People’s Planning has not reached every nook and corner of Kerala. Not all of our Medical Officers are that empowered to think about its seriousness…. When I was a Medical Officer, I never got a feeling that the department expected continuity of such work. (WS-3 Medical officer-3 female)

### C2: Communicable disease outbreaks and climate change-induced disasters reanimated community action in service of health and created a normative push towards interaction, and interdependence at the grassroots level

The initial momentum of decentralised planning seemed to settle in by late 2000 and then Kerala was struck by a string of outbreaks of Chikungunya and Dengue fever in the late 2000s, monsoon floods and landslides in the mid 2010s, the Nipah Virus outbreak in 2018, the COVID-19 pandemic 2020 onwards, with Zika virus cases reported in 2021. The state government managed to respond to these crises in a manner positively received by national and international media. A crucial element in these emergency responses was community participation and intersectoral collaboration with the support and leadership of LSGs. They have been the focal point in converging various government departments and communities to form a coordinated response to emergencies. A senior scholar-activist commented:

In 2010, there was a turnaround. For ten years, you could say that there was a period of a dark age for decentralisation, as one would say there was a dark age in Europe. This turnaround was specially brought about by developing a unique plan to intervene and control the epidemics in the Alappuzha district and its panchayats. It is from that point that the awards and recognitions started coming in. (WS-1 Academic-3 male)

A medical officer reiterated the role of epidemics and other communicable diseases in rekindling the spirit of decentralised health planning and interdepartmental convergence for improving health outcomes in Kerala, saying “The importance of public health was addressed at least to an extent with the coming of Chikungunya and some other communicable diseases. It opened up the possibility of convergence between various departments to face these crises.” (WS-3 Medical officer-2 male).

### M2: LSG leaders, frontline workers and other stakeholders collaborated and created grassroot level structures for health promotion through interdepartmental coordination

Post PPC in 1995; health department officials started working closely with LSG members for local health planning and implementing health programs utilizing LSG funds. The cooperation was crucial for the health department as LSG members also mobilized communities and facilitated interdepartmental coordination for various public health activities like cleanliness drives, source reduction for vector born diseases and improving community health literacy through well-established grass root level networks. 2006 onwards, N(R)HM further institutionalised the relationship between LSG members and health department actors by setting up Ward Health Sanitation and Nutrition Committees (WHSNC) and Hospital Development/management Committees (HDCs/HMCs). A key feature of NR(H)M to improve community action in health was to set up Rogi Kalyan Samitis (RKS) or patient welfare committees to enhance community voice in service delivery at public hospitals. In Kerala however, HDCs were already set up in all public health facilities with local community members and began to perform all the roles assigned to RKS in the state. A study participant who work with the health department and NHM remarked that functioning of HDCs/HMCs in Kerala may have inspired the whole concept of RKS itself:

The Government of India was very interested when they knew that we did all the RCH activities through HMCs. They were enquiring about how it has been done. An IAS officer told me at Trivandrum that a new project is coming up. We will be planning to replicate many of these activities in the new project throughout the country. (WS-3 NHM official 3 male)

Another salient feature of N(R)HM was the introduction of frontline female community health workers, or ASHAs with one worker per every 1000 persons, corresponding roughly to a panchayat ward population in Kerala. ASHAs worked along with an existing network of Anganwadi workers and Kudumbashree members. The state health department upskilled ASHAs based on the specific health system requirements of the state. A senior administrator involved in LSG policy making and planning attributed the effectiveness of the ASHA program in Kerala to their close links with LSGs:

If I look at the present COVID-19 situation, ASHAs are at the forefront at the local level, in the ward committees, because now they work together with the local government system and emerged as frontline warriors. This was not the case earlier, and ASHAs were kind of a particular system that was developed entirely through the N(R)HM but could not become the “healthcare activist” at the local level. Whereas now they have become the fulcrum of our activities simply because the system works together. (WS-1Policy maker-3 male)

In addition to the community health worker program, WHSNC were designed to be subcommittees of local self-government with around fifteen members, of which at least half were to be women. The ASHA was to be the chairperson and elected representatives (with preference given to women members), community members, and frontline health workers were to serve as members of the committee. The committees were set up to promote local health planning and increase the participation of communities in health. In Kerala, the health department, NR(H)M and LSGD department contributed funding to maintain and strengthen these community-level structures. As a senior health administrator who was involved with planning and policy pointed out:

Fund provision through NRHM was also a significant factor. For (WHSNC) there was NRHM fund provision of Rs. 10,000, additional Rs. 10,000 through Shuchitwa Mission, and Rs. 5000 from own fund of panchayats…N(R)HM untied fund was also started being given to sub-centres, PHCs and CHCs…In 2007, a revised Hospital Development Committee order was issued through the state NHM and RKS fund was made available. So, for the community-level interventions and programmes, there was some scope in the N(R)HM implementation framework. (WS-1 Policymaker-2 male)

### O2a: Multisectoral action at the grassroots level improved disease control and made health programs more inclusive

Decentralised health planning supported by LSG and N(R)HM at grassroots levels were described as initiating effective intersectoral action by many witnesses. Communicable and Non-Communicable Disease control often involves interventions in socio-economic determinants of health along with medical management of the disease by the health department. Providing such solutions is often outside the scope of the health department alone. The LSGs, with their autonomy, had the power to pool resources and schemes of different departments and implement solutions to local health issues reported by health workers. A medical officer cited two experiences of improved communicable disease control actiivties while working in a PHC in the Trivandrum district of Kerala:

Jaundice was rampant in one area. We (PHC staff) got to know it was an issue related to drinking water. To resolve that, a village committee was formed, and they raised this issue in the Grama Sabha meeting. As a result, realising the possibilities for integration, utilising the Rajiv Gandhi Drinking Water Scheme (implemented by the Kerala water authority), an overhead water tank was sanctioned. once the drinking water issue was resolved in that high-range area, not a single Jaundice case was reported.……… During the MDA (Mass Drug Administration program for Filariasis prevention), we (the PHC team) were able to make an 80% (drug) consumption rate because of people’s participation.(WS-3 Medical officer-2 male)

Local health planning at the LSG level could also ensure the inclusiveness of health programs. Elected representatives, with strong ties to the local community, were proactive about ensuring the inclusiveness of health services. An elected representative who served as a president in a panchayat narrated the role of decentralised planning in leaving no one behind:

We (LSGs) addressed issues of the older people, women, and children and challenges faced by differently-abled [sic.] people. In the case of physically challenged babies, we start thinking about physiotherapy only when it is time for schooling or maybe after five years of age. That is where LSGs can play a role. With decentralisation, we got to see and engage directly with such situations. I believe that is the biggest success story of decentralisation. There were a lot of limitations when things were decided from the top. But when decisions were taken from the bottom, we were able to enumerate the number of differently-abled children [sic], understand what were their challenges, what are their health issues, what of all those issues can we intervene and give a solution [for] etc. Thus, we were able to prioritise and allocate sufficient funds required for that. (WS-2 Elected representative 1 female)

The state government used NR(H)M resources to support the continuation of small stand-alone programs designed to meet the needs of the marginalised sections of communities in selected geographies. An example was a bespoke program benefiting indigenous communities who face a disproportionate burden of sickle cell anaemia (an inherited blood disorder) in the state:

My personal experience in the case of Wayanad and Attappady’s comprehensive sickle cell care project is worth mentioning. This was a project funded by the European Union for one year. But for the subsequent support from the NRHM, this project would never have gone forward. Even today, a unit of the project is functioning in Calicut Medical College and the funds of NRHM support the work in Wayanad and Attappady. (WS-1 Academic-2 male)

The spirit of PPC induced coordination across stakeholders was also observed in the pandemic. As observed by an academic “everyone came together in one platform… when COVID-19 hit us in 2020; by then department of health services, medical colleges, community volunteers, LSG’s grama panchayat and block panchayat were all aligned in one platform.” (WS-3 Academic-4 female).

### O2b: Avenues emerged for community health workers (ASHAs) to assume wider roles in the health sector and take up leadership roles in local governance institutions

The range of roles performed by ASHAs in Kerala has expanded considerably over the years.A senior policy maker noted this transition:

The pleasant surprise was the functioning of ASHA workers, whom initially many thought would be redundant in Kerala, because the State had already a good front line system in health. Mainly due to the push from local governments, ASHA workers have carved out a niche area at the cutting edge level (WS-1 Policymaker-1 male).

A senior N(R)HM official who managed the ASHA program from 2007, added that the state health department delegated NCD control and palliative care to ASHAs early on rather than restricting their work to maternal and child care services for which the program was initially designed:

Another main linkage with the LSG was the ASHA program that we started in 2007.….Their activities are fully linked to the panchayats. In today’s scenario, Kerala is the only state where ASHA workers address all the issues related to non-communicable diseases, palliative care, mental health, etc. (WS-3 N(R)HM official-3 female)

The work ASHAs have done over the years with the community and the close working relations they maintained with the LSGs has led many to consider running for and getting elected an LSG member. This fostered grassroot level female leadership in Kerala. A female N(R)HM official remarked “during this election (Kerala LSG election-2021) 746 people (ASHAs) got elected, 113 of them are Presidents, health standing committees and different standing committees.” (WS-3 N(R)HM official-4 female).

## Discussion

In this study, we analysed data from three Witness Seminars exploring decentralisation or PPC reforms in the N(R)HM era in Kerala using a realist approach. We found two CMO configurations to explain the nature of the interaction between PPC and N(R)HM. CMO 1 describes that decentralised health planning in Kerala during the late nineties had a positive impact on Kerala’s health system. It opened avenues for innovative health programs at the grass roots level. The State health department scaled up selected LSG projects like palliative care across the state. The decentralisation efforts and emergence of N(R)HM introduced structural improvements in HRH, medicine supply, infrastructure, and improved service quality. Over the years, institutionalisation, digitisation, and the limited capacity of implementors emerged as the major challenges in sustaining these innovations. The second CMO configuration suggests that after the initial years post-PPC in 1995, the momentum settled, and the emergence of climate change-induced disasters and disease outbreaks rekindled community participation in health under the leadership of LSGs. The process of decentralisation and the transfer of ownership of PHCs to LSGs and N(R)HM created more stakeholders like ASHA and grassroots-level structures like WHNSC. Over the years, ASHA’s have emerged as a strong accepted cadre of community health workers and many of them ventured to electoral politics and assumed leadership positions in LSGs strengthening the grass root level democracy.

These cross linkages– between people’s participation, service delivery, and multisectoral action are well established as being the core of primary health care as per the World Health Organization’s primary health care performance framework [30]. The ‘Kerala model,’ although quite highly privatised and medicalised at present, may nonetheless offer some entry points to understand the synergies of these linked processes.

Our analysis joins the canon documenting innovative health programs that came as a result of decentralised health planning [10, 31] as well as literature on the impact of N(R)HM in the Kerala health system [32, 33]. Our study participants reflected that empowering LSGs led to many innovative health projects in Kerala answering to the varying health needs of local communities. Elamon et al., in a study done in 2004, also describe this in detail, noting that around 150 innovative projects were developed during the five years of the campaign by 1,214 local councils of Kerala. As observed by our participants, these innovative projects ranged from mosquito control projects to setting up model healthy villages and cooperative secondary care facilities [10]. Published literature on the role of N(R)HM in increasing the fund allocation from LSGs through programs like comprehensive health plans was not available. But the impact as mentioned by our study participants could be corroborated by data from a presentation made by the social development wing NHM Kerala accessible from the archives of India’s National Health Systems Resource Centre, or NHSRC [34].

The current study found that the synergy of decentralisation initiatives starting in the mid-90s and N(R)HM from the mid-2000s strengthened Kerala’s public sector health systems building blocks. These findings are consistent with the existing literature, which records the construction and renovation of existing public health infrastructure and staff postings done by LSGs [35–37]. The second Common Review Mission in 2008 on NRHM performance in Kerala also noted the marked improvement in HRH and health infrastructure of the health institutions by utilizing N(R)HM funds [38].

A critique of the decentralised planning process in health from our study was that gradual institutionalisation of the process has hampered innovation after a certain point as additional fund allocations for innovations were not provided and existing funds were earmarked to ongoing programs. A recent study in Poland which looked at the role of local and regional authorities in NCD care also documented similar finding [39]. Our study finds that constitutional amendments to decentralise planning and the PPC initiative improved the decision space, in health planning i.e., the choices allowed by a central authority to the local authority to utilise. However, it is riddled with tendencies of re-centralisation of planning and power relation with central agencies,similar experiences are documented in Pakistan, the Philipines and Uganda, with experience of decentralised health planning [40–42]. Bossert et al. proposed a framework to study decision space by looking at the choices offered by central agencies to local institutions, the discretionary power exercised by local authorities, and the impact it has on improving health outcomes [43]. Seshadri et al. used this framework to study NRHM and its role in decentralisation in Karnataka India and found that though the guidelines for decentralisation remain the same the uptake and use of power by local authorities differed across districts [13]. The 72 and 73rd constitutional amendments for devolution of power were national mandates, but the uptake by states in India differed substantially. Decentralisation reforms were implemented with a certain degree of success in Kerala compared to other states due to, firstly, the long history of progressive movements, which included land redistribution reforms, mass science awareness, and literacy campaigns, women’s empowerment, struggles against the caste system and untouchability, to which the PPC was a natural continuation. Secondly, the strong and sustained political will from early nineties onwards has been evident from continuous support to the process and subsequent institutional mandates to devolve 40% of state plan fund to LSGs [44] and to set up the Kerala institute for Local Administration (KILA)a technical unit, to handhold the process [45].

Our study notes the scale-up of the palliative care movement, which started as a voluntary project on a small scale in panchayats of Northern Kerala which was supported by LSGs, and it was further expanded across the state with the support of the state government and N(R)HM. Kumar et al. further describe the history of palliative care initiatives in Kerala and note the transition of palliative care from a doctor-led program approach to a community-owned initiative [46]. The mechanism of LSGs and N(R)HM complementing and supporting the palliative care program is also noted by Rajeev et al., who also pointed out some critical weaknesses in the program implementation like poor quality of service, lack of training for staff and key stakeholders, and more [47]. The strengths and weaknesses of this program warrant greater study and policy attention.

The occurrence of communicable disease outbreaks and climate change-induced disasters in Kerala and the role of community action and intersectoral convergence mechanism as a key element in managing these adverse health events reported by our study is consistent with studies conducted by Sudeep et al. and Joseph et al. [48, 49]. The findings of KC et al. resonate with ours, reflecting positively on the role of LSGs and frontline health workers in managing the COVID-19 pandemic. [50] Mathew et al., in their comprehensive analysis looking at the role of LSGs in disaster management, also report the role of institutionalised structures nurtured by PPC and N(R)HM in the community, which slowly evolved to become the cornerstone of public health activities in the state [51].

During the 2020 LSG elections in Kerala, 774 ASHAs were elected, of which 31 assumed presidency, 31 assumed LSG president posts and 123 ASHAs were part of various standing committees [52]. As regards to ASHA honoraria, these remain uniform throughout the country; the allocation split between central and state governments. While the LSG candidacy offers a career trajectory to ASHAs, their honorarium of Rs 10,000 (USD120) remains suboptimal in the state [53–55]. Kerala’s traditionally robust education system improved levels of women’s education, and state-sponsored empowerment initiatives like kudumbhasree equipped the ASHAs to participate in the electoral process and acquire leadership positions at various levels of the panchayat system [56, 57]. The progression of community health workers into broader leadership roles has been seen in the case of Chhattisgarh’s progenitor program of Mitanin (which preceded the national ASHA program) [26] and suggests that greater attention to and support of these actors in the system will be necessary going forward. Needs and priorities may be identified through further research in the state, particularly given the unique and multifarious roles played by ASHAs in and beyond disaster contexts.

### Strengths and limitations

Our study used a realist approach to analyse the experiences of participants (i.e. witnesses) about events they were part of, revealing implementer perspectives that were previously undocumented. The method of the WS is a co-creation of history and provides a robust reference to nuances of program history. The major limitation of the study was the lack of documentation available to support some of the events, programs, and facts mentioned by Witnesses, there was either little documentation or lack of documentation– online or even in archives and libraries. This points to the importance of recording history before it completely goes unrecorded. Witness Seminars are an effective way to document history, as they provide ample information on events with a general agreement from the participants attending the seminar. While the transcripts of the seminar offer an overall view, it may sometimes be necessary to collect additional data to gain a deeper understanding of certain events, particularly from the perspective of those who may be targeted or intended beneficiaries of programmes, but not directly involved in their development or production. The outcomes identified in our study are constructed by arranging expert opinion and they could be further studied through economic evaluations or realist evaluations. Another limitation of our Witness Seminars is that they were held virtually, which placed some constraints of communication and time boundedness. Were they to be carried out in person, we perhaps would have different and additional insights. This is planned in our future research on community action for health.

## Conclusion

Decentralised planning and N(R)HM have made significant contributions to the health sector by way of innovative, inclusive and intersectoral programming, responsive to local needs, that added value to core health system building blocks in a southern Indian state. This was enhanced in the context of emergencies and disasters. However, centralised health planning still belies the ethos and imperative of decentralisation and a careful balance likely needs to be struck between central(ised) programmes and decentralised governance in and beyond the health sector, particularly as frontline workers enter the fray of leadership across these institutional mechanisms.

## Data Availability

The full dataset generated or analysed during the current study is available at: https://www.georgeinstitute.org.in/our-impact/policy-and-recommendations/community-action-for-health-evidence-from-india
